# Biological and Molecular Characterization of a New Isolate of Tomato Mottle Mosaic Virus Causing Severe Shoestring and Fruit Deformities in Tomato Plants in India

**DOI:** 10.3390/plants13192811

**Published:** 2024-10-08

**Authors:** Prantik Mazumder, Firoz Mondal, Mehulee Sarkar, Anik Majumdar, Kajal Kumar Biswas, Susheel Kumar Sharma, Milan Kumar Lal, Rahul Kumar Tiwari, Ravinder Kumar, Anirban Roy

**Affiliations:** 1 Advanced Centre for Plant Virology, Division of Plant Pathology, ICAR-Indian Agricultural Research Institute, New Delhi 110012, India; mazumderprantik@gmail.com (P.M.); mdfirozmondal1997@gmail.com (F.M.); mehulee2014@gmail.com (M.S.); anikmajumdar5757@gmail.com (A.M.); drkkbiswas@yahoo.co.in (K.K.B.); susheelsharma19@gmail.com (S.K.S.); 2 ICAR-National Rice Research Institute, Cuttack 753006, Odisha, India; milan2925@gmail.com; 3 ICAR-Indian Institute of Sugarcane Research, Lucknow 226002, Uttar Pradesh, India; rahultiwari226@gmail.com

**Keywords:** shoestring, fruit deformities, tomato mottle mosaic virus (ToMMV), tobamovirus, host range, seed transmission

## Abstract

Tomato (*Solanum lycopersicum* L.), the second most important vegetable crop globally, faces a significant threat from various viral diseases. A newly emerging disease, characterised by distinctive shoestring symptoms on leaves and the development of unripe, small, and hard fruit, poses a serious challenge to tomato cultivation in India. An initial survey in an experimental field revealed more than 50% of the plants displayed symptoms of the shoestring disease, resulting in substantial reductions in fruit yield and quality. Transmission electron microscopy (TEM) and molecular analyses identified an isolate of the tomato mottle mosaic virus (ToMMV) in the affected plants. When the partially purified virus was mechanically inoculated into tomato cv. Pusa Ruby plants, it reproduced the characteristic shoestring symptoms, confirming its causal relationship with the disease. Notably, the present shoestring isolate of ToMMV (ToMMV-Tss) was found to induce similar shoestring symptoms in most of the major commercial tomato varieties when inoculated under controlled experimental conditions in the glasshouse, indicating its aggressive nature. Host range studies demonstrated that the ToMMV-Tss can infect several solanaceous species, while cucurbitaceous hosts remained unaffected. Moreover, the virus was found to be seed-transmissible, with a small percentage of seedlings from infected plants displaying symptoms. These findings underscore the significant impact of ToMMV on tomato production in India and emphasise the need for reliable diagnostic tools and effective management strategies to curb the spread and mitigate the impact of this virus on commercial tomato cultivation.

## 1. Introduction

Tomato (*Solanum lycopersicum* L.) is the second most important vegetable crop globally. Like other economically important crops, tomato plants are also vulnerable to many diseases caused by fungi, bacteria, and viruses. Around 146 viruses from 33 different genera are known to infect tomato plants worldwide [1]. The major viral diseases affecting the crop include leaf curl, mosaic, wilt, and necrosis. Recently, a shoestring disease has emerged as a serious threat to tomato production in several commercial cultivars of tomato in the major tomato-growing regions of the country. The affected plants exhibited significant leaf deformities, often resulting in the formation of tendril-like structures. The disease initially manifested as mild mosaic symptoms on the leaf, which subsequently progressed to severe leaf shoestring symptoms. Additionally, the affected fruits exhibit deformities and hardness with reduced size, significantly impacting the marketability of the produce. The disease has become the headline in all the leading newspapers due to the skyrocketing price of tomatoes in the Indian market. Unconfirmed reports have suggested a potential association of cucumber mosaic virus (CMV) (species: *Cucumovirus CMV*, genus: *Cucumovirus*, family: *Bromoviridae*), tobamovirus(es), and begomovirus(es) with the disease. Furthermore, reports have highlighted the distress of farmers who have constantly complained about the deteriorated quality of the fruits in terms of size, colour, and firmness. Shoestring symptoms in tomato plants have been linked globally either to CMV or to different tobamoviruses (family *Virgaviridae*). In India, shoestring symptoms in tomato plants were noticed earlier in other regions of India with lower incidence. A distinct isolate of CMV was reported to be associated with the disease [2,3,4,5]. However, none of these studies indicated the hardness and deformities of the tomato fruits. A similar shoestring disease without the hardness of the tomato fruits was also documented in Chile. The causative agent was reported to be an isolate of tomato mosaic virus (ToMV) [6]. On the other hand, recently, an isolate of tomato brown rugose fruit virus (ToBRFV) was reported to cause hardness of fruit with brown patches but without any shoestring symptoms [7]. As this new disease of tomato plants displays distinct symptom phenotypes, such as a shoestring appearance of leaves and hard, deformed fruits, the present study aimed at uncovering its aetiology by biological and molecular analysis.

## 2. Results

### 2.1. Natural Occurrence of Shoestring Disease in Tomato cv. Pusa Ruby

**Figure 1:**
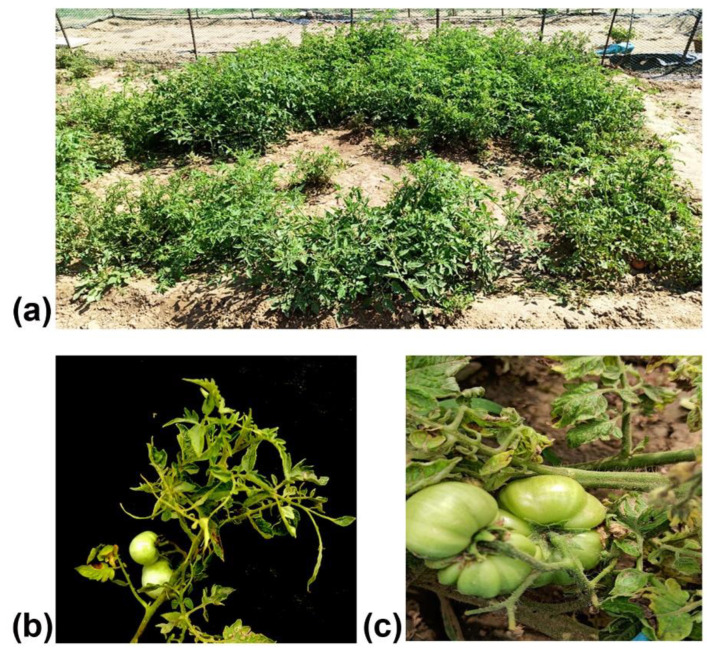
Occurrence of shoestring symptoms in tomato cv. Pusa Ruby in the IARI experimental field. (**a**) Disease affected tomato field, (**b**) shoestring symptoms in tomato cv. Pusa Ruby plants, (**c**) deformed and hard unripe fruits.

A survey was conducted in an experimental field of the Division of Plant Pathology, Indian Agricultural Research Institute during autumn 2023 (September–October) to document the incidence of different viral diseases in the tomato cv. Pusa Ruby plants. The records showed that in more than 50% of the plants, the leaves were tendril-like with very narrow leaf lamina, resulting in a shoestring-like appearance (Figure 1a,b). Besides such symptoms, green mosaic and chlorotic mottling symptoms were observed in ~20%, and leaf curl symptoms in ~20% of plants. The majority of the shoestring-affected plants did not bear any fruit. However, in the plants where fruit sets were observed, fruits were hard, deformed, irregularly shaped, and reduced in size (Figure 1c). The severely infected plants were stunted and exhibited reduced growth.

### 2.2. Association of an Isolate of Tomato Mottle Mosaic Virus with the Shoestring Symptom in Tomato as Evidenced by TEM, RT-PCR, and Sequencing

**Figure 2:**
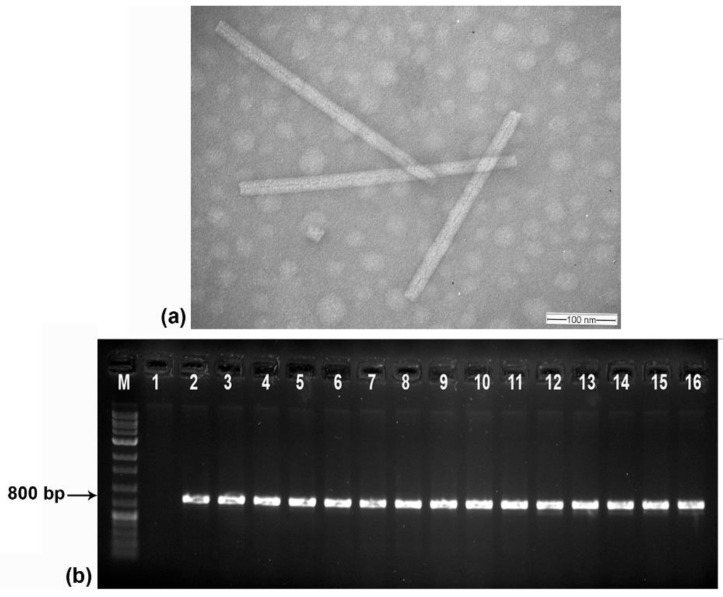
Confirmation of the association of a tobamovirus with shoestring symptoms in tomato plants in India through transmission electron microscopy and reverse-transcriptase PCR (RT-PCR). (**a**) Representative transmission electron micrograph using 2% uranyl acetate (negative stain) of infected leaf samples under 80,000× magnification showing the presence of rigid rod particles (~300 × 18 nm). (**b**) Agarose gel (1%) electrophoresis of the ~800 bp RT-PCR-amplified product obtained from all the symptomatic samples tested using tobamovirus generic primers. M: molecular weight marker; 1: negative control; 2: positive control; 3 to 16: field samples.

**Figure 3:**
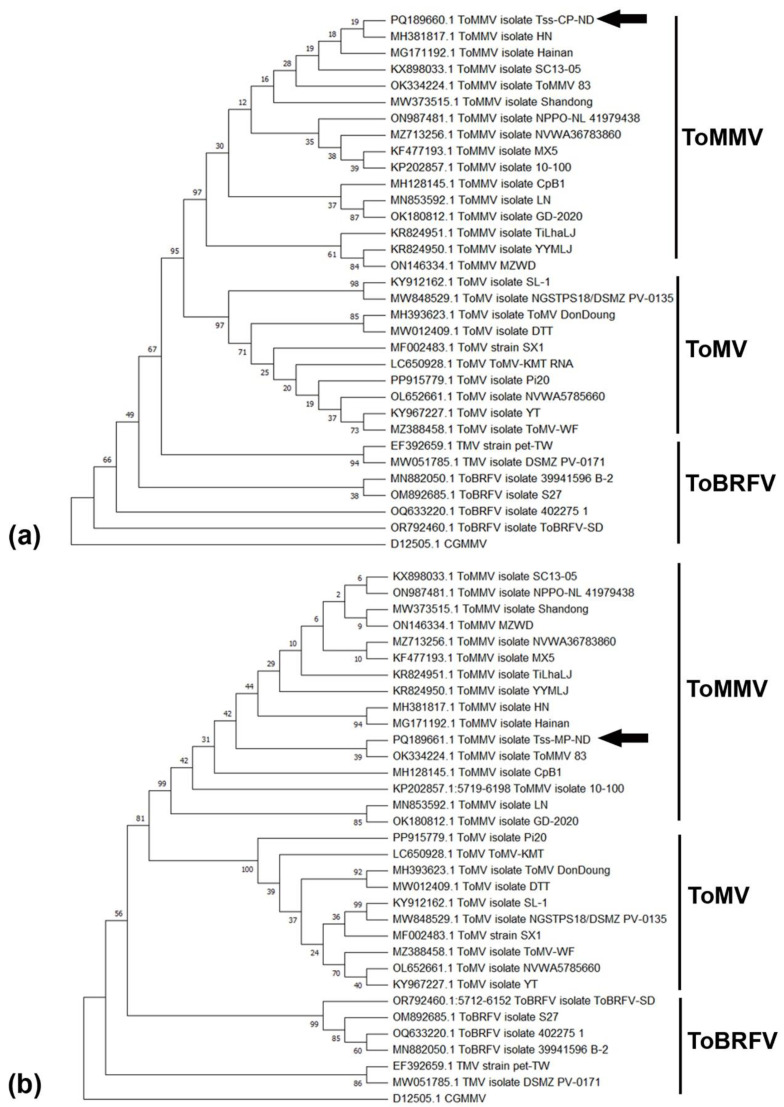
Phylogenetic relationship of the CP (**a**) and MP (**b**) genes of ToMMV-Tss with other isolates and other tobamoviruses. The dendrogram was constructed using the neighbour-joining method in MEGA11. A bootstrap analysis with 1000 replicates was performed. Black arrows mark the ToMMV-Tss.

Transmission electron microscopic (TEM) analysis revealed the presence of tobamovirus-like rigid rod-shaped particles with a size of ~300 X 18 nm in all the symptomatic samples (Figure 2a). However, CMV-like spherical particles (28–30 nm) were not detected in any of the symptomatic samples tested. Subsequently, direct antigen-coated ELISA (DAC-ELISA) using a CMV coat protein-specific polyclonal antibody (produced earlier in our laboratory) revealed none of the test samples produced a positive reaction, indicating the absence of CMV in the tested samples. The OD values of the positive control samples were 2 to 3 times higher than those of the healthy/buffer controls (negative control) (Figure S1a, Table S1). Further, to confirm the association of a tobamovirus and also to validate the absence of CMV in the symptomatic samples, reverse transcriptase PCR (RT-PCR) analysis was carried out using universal tobamovirus generic primers and different primers specific for CMV, TMV, ToMV, and ToBRFV (Table S2). All 12 tested field samples yielded the expected amplicon with tobamovirus generic primers (Figure 2b). None of the samples had any amplification with either the CMV-specific primers or other tobamovirus species-specific primers tested (Figure S1b–g). The RT-PCR-amplified product obtained using tobamovirus generic primers was cloned and sequenced. NCBI BLASTn analysis of the nucleotide sequence of the cloned DNA showed >98% sequence identity with different isolates of tomato mottle mosaic virus (ToMMV). The sequenced region spans the complete ORF of the coat protein (CP) gene and the partial ORF of the movement protein (MP) gene. Subsequently, primers for specific amplification of complete CP (AR47F and AR48R) and MP (AR51F and AR52R) genes were designed and these two genes were amplified (Figure S2), cloned, and sequenced. In multiple sequence alignment, the CP sequence (Acc. no. PQ189660) exhibited a range of 89.9–99.5% and 98.7–99.3% identity percentages with other ToMMV isolates at the nucleotide and amino acid levels, respectively (Table 1). Likewise, the MP sequence (Acc. no. PQ189661) displayed a range of 98.6–99.3% and 98.5–99.6% identity percentages with other ToMMV isolates at the nucleotide and amino acid levels, respectively. However, in comparison with other tobamoviruses, the CP sequence showed a range of 44.9–85.6% and 32.9–91.1% identity percentages at the nucleotide and amino acid levels, respectively, and the MP sequence exhibited a range of 42.2–77.4% and 28.3–76.8% identity percentages at the nucleotide and amino acid levels, respectively. Phylogenetic analysis with both CP and MP sequences revealed that the present ToMMV-Tss (ToMMV-Tss) was grouped with other ToMMV isolates reported from tomato plants (Figure 3).

### 2.3. Mechanical Inoculation of Partially Purified ToMMV-Tss Produced Typical Leaf Shoestring Symptoms in the Tomato cv. Pusa Ruby Plant

**Figure 4:**
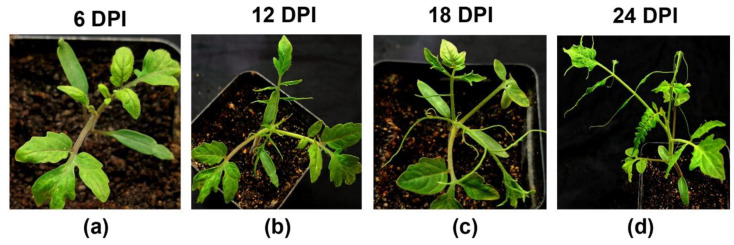
Symptom progression in tomato variety Pusa Ruby plants after inoculating a partially purified shoestring isolate of ToMMV (ToMMV-Tss). (**a**–**d**) The progression of disease at 6, 12, 18 and 24 days post inoculation (DPI).

The virus was partially purified and checked with TEM (Figure S3). Mechanical inoculation of the partially purified virus on tomato cv. Pusa Ruby plants produced mild mosaic symptoms within 6–8 days post inoculation (dpi). Within 12 dpi, newly emerging leaves of the inoculated plants started appearing narrow, with mild downward leaf curling and stunted growth. The distinctive shoestring symptom was observed around 15–18 dpi. Within 24 dpi, severe shoestring of leaves and leaf deformities were recorded (Figure 4). Fruit deformities could not be recorded as the plants died due to the high severity of the disease before the flowering stage.

### 2.4. Most Commercial Tomato Cultivars Are Susceptible to the ToMMV Isolate

**Figure 5:**
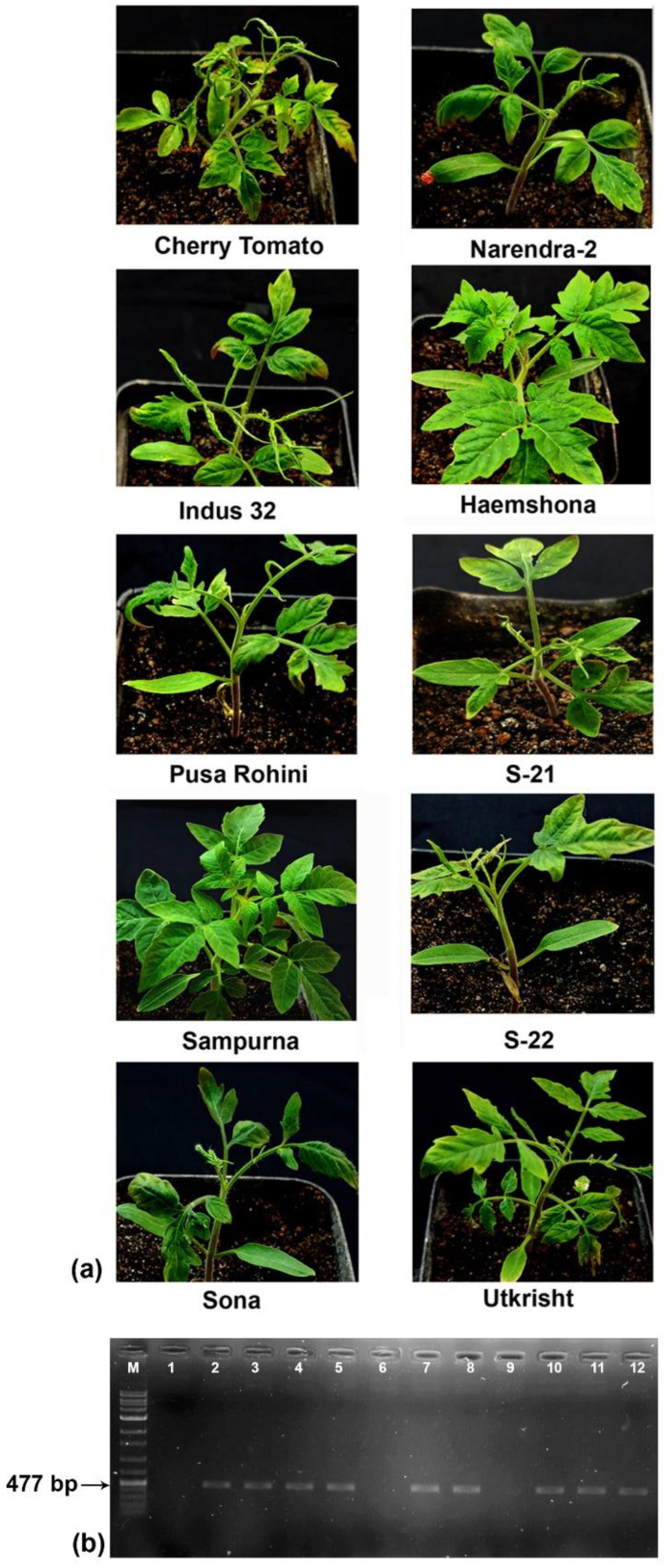
Response of different tomato cultivars upon mechanical inoculation of a partially purified ToMMV-Tss. (**a**) Symptom development was noticed in eight cultivars while two cultivars, Haemshona and Sampurna, did not exhibit any symptoms. (**b**) Agarose gel (1%) electrophoresis of the RT-PCR-amplified coat protein gene of the ToMMV-Tss using specific primers from the ten inoculated cultivars. The absence of amplified products in the tomato cultivars Haemshona (Lane 6) and Sampurna (Lane 9) indicated their resistance against ToMMV-Tss. Lane 1: healthy control; lane 2: positive control; lanes 3–12: tomato cultivars Cherry tomato, Narendra 2, Indus 32, Haemshona, Pusa Rohini, S-21, Sampurna, S-22, Sona, and Utkrisht.

Eight of ten commercial tomato cultivars inoculated with the partially purified virus exhibited characteristic mosaic and shoestring symptoms in 15–20 dpi. The cultivars Haemshona and Sampurna remained asymptomatic after 28 dpi (Figure 5a). Detection of the virus using the AR47F and AR48R primers showed an expected amplification of ca. 477 bp from the symptomatic plants of the eight cultivars (Figure 5b). However, Haemshona and Sampurna’s cultivars did not yield any amplification indicating these cultivars may have resistance against the virus.

### 2.5. ToMMV-Tss Causes Disease in Solanaceous Hosts but Not in Cucurbits

**Figure 6:**
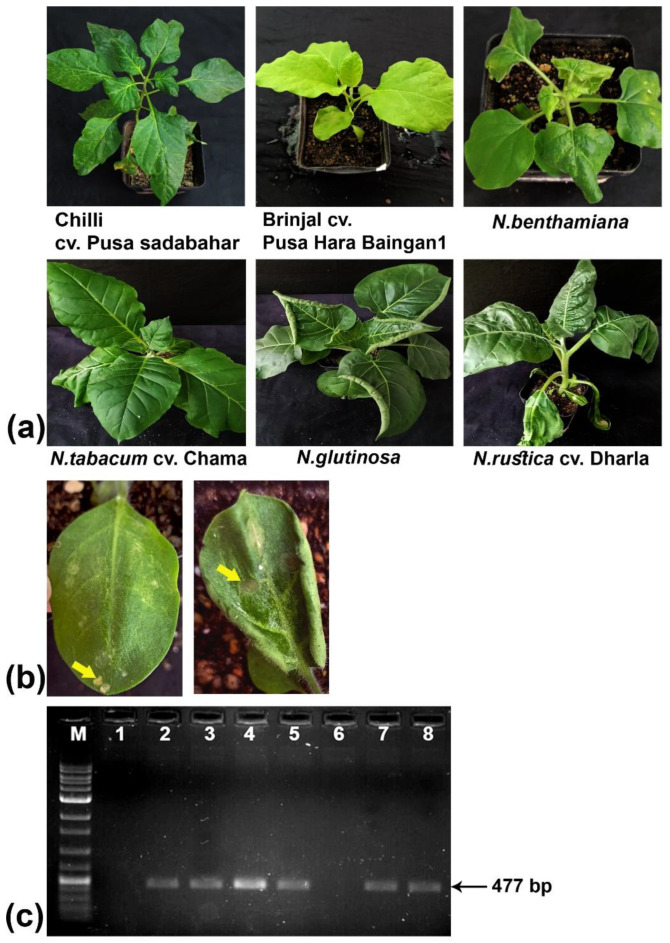
Responses of different hosts under the family *Solanaceae* upon mechanical inoculation of partially purified ToMMV-Tss. (**a**) Symptom phenotype in the inoculated plants of chilli, brinjal (no symptoms), *N. benthamiana*, *N. tabacum* (no symptoms), *N. glutinosa* and *N. rustica.* (**b**) Local lesions observed (yellow arrow) in *N. rustica* (**left**) and *N. glutinosa* (**right**). (**c**) Agarose gel (1%) electrophoresis of the RT-PCR-amplified coat protein gene of the ToMMV-Tss using specific primers from the six inoculated solanaceous plants. The absence of the amplified product in *N. tabacum* cv. Chama (Lane 6) indicated their resistance against ToMMV-Tss. Lane 1: healthy control; lane 2: positive control; lanes 3–8: chilli, brinjal, *N. benthamiana*, *N. tabacum*, *N. glutinosa*, and *N. rustica*.

Out of the six different plant species within the *Solanaceae* family, including four experimental hosts of tobacco and its wild relatives, chilli plants exhibited systemic green mosaic symptoms after three months, but none of the brinjal plants inoculated with the partially purified ToMMV exhibited any symptoms (Figure 6a, Table 2). However, in RT-PCR amplification using CP-specific primers, inoculated brinjal samples produced the expected amplification, indicating the cultivar of brinjal used may show asymptomatic behaviour upon inoculation with ToMMV-Tss. Among the *Nicotiana* species included in the study, *Nicotiana benthamiana* plants exhibited downward curling of leaves, systemic puckering, blistering symptoms, and severe stunted growth (Figure 6a, Table 2). *Nicotiana glutinosa* plants initially showed local necrotic lesions and marginal upward rolling of systemic leaves at a later stage (Figure 6b, Table 2). *Nicotiana rustica* cv. Dharla also exhibited necrotic local lesions at the initial stage and systemic puckering symptoms later (Figure 6b, Table 2). However, *Nicotiana tabacum* cv. Chama plants showed no symptoms following mechanical inoculation with partially purified ToMMV-Tss. Except for *Nicotiana tabacum* plants, all the other plants gave positive amplification with the coat protein-specific primers (Figure 6c, Table 2). The partially purified virus neither produced any symptoms in the seven cucurbitaceous hosts tested, nor showed any positive amplification (Figure S4).

### 2.6. Shoestring Disease Causing ToMMV-Tss in Tomato Plants Is Transmitted by Seeds

**Figure 7:**
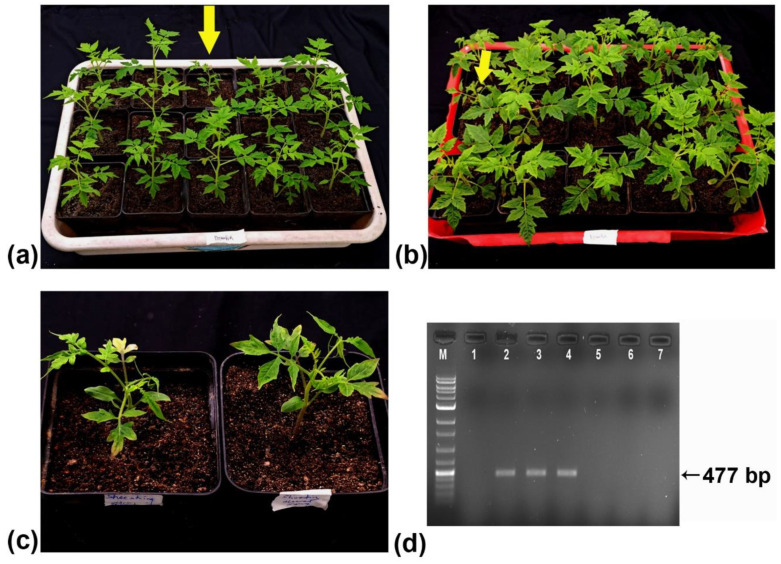
Seed transmission behaviour of the ToMMV-Tss. (**a**,**b**) Germinated seedlings grown from the seeds of virus-infected tomato (cv. Pusa Ruby) plants, of which two plants showed shoestring symptoms (marked by yellow arrow). (**c**) Symptomatic plants grown from the infected seeds. (**d**) Agarose gel (1%) electrophoresis of the RT-PCR-amplified coat protein gene of the ToMMV-Tss using specific primers from five plants, including two plants showing symptoms. Lane 1: healthy control; lane 2: positive control; lanes 3–4: symptomatic plants; lanes 5–7: asymptomatic plants.

To ascertain the seed transmissibility of ToMMV-Tss causing shoestring disease in tomato plants, fifty germinated seedlings grown from the seeds of the infected plants were regularly checked for the emergence of symptoms. After four weeks, the leaves of two of the seedlings (4%) exhibited mosaic and shoestring symptoms, which progressed to severe leaf deformation at the later stage (Figure 7). The infected seedlings exhibited stunted growth in comparison to the healthy seedlings.

## 3. Discussion

The main aim of this study was to determine the aetiology of a recently emerging severe shoestring disease of the tomato crop in India. The high incidence of shoestring disease in an IARI experimental field underscores the high susceptibility of one of the important commercial tomato cultivars, Pusa Ruby, to viral infections under field conditions. Typical symptoms of the disease include severe reduction of leaf lamina with shoestring appearance, stunted growth, and production of small, hard, unripe deformed fruits, and under severe conditions, complete failure of fruit setting, suggesting a profound impact of the disease on crop yield and marketable quality. Earlier studies showed shoestring symptoms in tomato plants caused by an isolate of CMV [2] from the Andhra Pradesh state and Lucknow region of the Uttar Pradesh state [3,4]. In another study, the RNA3 genomic component of CMV associated with shoestring disease from Delhi was fully sequenced, revealing the presence of a unique strain of CMV called Tss-In [5]. In recent years, there has been a sudden surge in the incidence of shoestring disease which also showed fruit deformities in most tomato-growing areas, leading to a decline in tomato production. To investigate the cause of the disease, we initially utilized transmission electron microscopy (TEM), which revealed the presence of a rigid, rod-shaped virus similar to tobamoviruses. In contrast, none of the samples tested showed the presence of CMV. The identity of the tobamovirus associated with the disease and the absence of CMV was further confirmed by ELISA using CMV-specific antisera and through RT-PCR amplification using specific primers for CMV and different tobamoviruses. The results revealed that none of the infected samples had CMV, TMV, ToMV, or ToBRFV. Furthermore, an expected amplification with generic tobamovirus primers and its sequence analysis revealed the tomato shoestring disease was associated with an isolate of ToMMV (ToMMV-Tss), which was not reported earlier in India. ToMMV is a member of the species *Tobamovirus maculatessellati*, containing a positive-sense RNA genome under the genus *Tobamovirus* (family *Virgaviridae*) (https://ictv.global/taxonomy/, accessed on 24 August 2024). It shares characteristics with other *Tobamovirus* species, such as the tobacco mosaic virus (TMV), tomato mosaic virus (ToMV), and tomato brown rugose fruit virus (ToBRFV). All these tobamoviruses cause mosaic, mottling, deformities in tomato leaves, brown patches in fruits, and stunted growth of infected plants. Tobamoviruses are transmitted through mechanical contact, propagation materials, plant debris, contaminated soil, growing media, circulating water, farming activities of workers, and shared cultivation tools [8,9]. Our findings of the association of a rigid rod-shaped virus, typical of tobamoviruses, align with findings by Li et al., 2013 [10], which first identified the associated tobamovirus as ToMMV in symptomatic tomato plants in 2013 from Mexico. Subsequently, it has been identified in the USA, Israel, Spain, China, Brazil, Australia, the Netherlands, and France [11,12,13,14,15,16,17,18]. However, as per the recent EPPO Global Database (https://gd.eppo.int/taxon/TOMMV0/distribution/ES, accessed on 24 August 2024), ToMMV, which was reported from seed lots in France and Spain, has not spread further. Therefore, ToMMV was declared absent from France and Spain. In earlier studies, it was reported that ToMMV causes mosaic, mottle, narrowing, and crinkling symptoms on the leaves of tomato plants, along with necrosis in the fruit [14,19], leading to stunted growth and reduced fruit yield. However, in the present study, shoestring leaves and hard unripe small tomato fruits are the most predominant symptoms under field conditions. The inability to detect any CMV and other tobamoviruses such as ToMV, TMV, or ToBRFV through RT-PCR suggests that under field conditions, the symptoms of the disease are mainly contributed by ToMMV. Comparison of the CP and MP sequences of the ToMMV-Tss with other ToMMV isolates and different tobamoviruses revealed a high degree of sequence identity at both the nucleotide and amino acid levels among all the ToMMV isolates. These findings are consistent with previous studies that have reported relatively low genome diversity for all known ToMMVs [19,20], supporting the hypothesis of the recent emergence of this novel virus [10]. Phylogenetic analyses of the CP and MP sequences of the ToMMV-Tss strongly support the classification of this new isolate within the subgroup of ToMMVs that infects solanaceous plants. 

In controlled experimental conditions, inoculation of the partially purified ToMMV-Tss in the tomato cultivar Pusa Ruby initially caused mild mosaic symptoms. Subsequently, it led to the characteristic shoestring symptoms on leaves, confirming its causal relationship to the shoestring disease in tomato plants. The present study thus not only expands the known geographic boundary of ToMMV, but adds a new symptom phenotype caused by it. Like the tomato cultivar Pusa Ruby, under controlled experimental conditions, most of the key commercial tomato varieties grown in India also displayed typical shoestring symptoms, highlighting the growing acknowledgment of ToMMV as an emerging virus impacting tomato cultivation. Two cultivars, Haemshona and Sampurna, neither exhibited any symptoms nor tested positive in RT-PCR, indicating their resistance behaviour. However, a more detailed study with resistant gene markers will be required in the future to establish their resistance behaviour conclusively. Additionally, the seed transmissibility of the ToMMV-Tss aligns with the earlier findings [16,17], which reported similar seed transmission rates for ToMMV, emphasising the importance of seed health in managing the spread of this virus. However, the relatively low seed transmission rate observed suggests that while ToMMV can be seed-borne, the major transmission of the virus predominantly occurs through mechanical means. The host range analysis revealed that the ToMMV-Tss can infect multiple species within the *Solanaceae* family, including chilli, brinjal, and different species of *Nicotiana*, while *N. tabacum* cv. Chama and all the cucurbit hosts remained unaffected. Earlier reports also suggested that ToMMV caused natural infection to tomato (*Solanum lycopersicum* L.) and pepper (*Capsicum annuum* L.) [20,21] but not to any cucurbits [19]. However, a recent report indicated natural infection of ToMMV in Chinese sneak gourd (*Trichosanthes kirilowii*) [22]. Regarding the infection of brinjal and *N. tabacum*, different researchers reported differently, most probably due to the differential response of different cultivars they tested [14,19,23]. In this study, though none of the brinjal plants produced any symptoms, positive amplification indicated the presence of the virus in the inoculated plants. Such asymptomatic plants could serve as reservoirs of the virus and help spread the virus to other crops. ToMMV was also reported to cause natural infection in leguminous crops like chickpea (*Cicer arietinum* L.) [24] and pea (*Pisum sativum* L.) [25]. Besides these hosts, under experimental conditions, plants in the families *Brassicaceae* and *Amaranthaceae* are also susceptible to systemic infection by ToMMV [14,20]. 

## 4. Materials and Methods

### 4.1. Field Survey and Collection of Infected Leaf Samples

A survey was conducted during September–October 2023 in an experimental field of the tomato (cv. Pusa Ruby) crop at the Division of Plant Pathology, ICAR-Indian Agricultural Research Institute, Pusa, New Delhi to understand the occurrence of viral diseases. Tomato leaves exhibiting shoestring symptoms were collected for further investigation regarding the causal agent of the disease.

### 4.2. Electron Microscopy

The presence of virus particles was examined in a transmission electron microscope (JEM 1011, JEOL USA, Inc, Peabody, MA, USA) using a leaf dip procedure at the Advanced Centre for Plant Virology, Division of Plant Pathology, IARI, New Delhi. A grid was prepared using negative stain uranyl acetate (2% aqueous, pH 4.2) and after a few minutes of air drying, the grid was finally viewed under a transmission electron microscope at 80,000× magnification.

### 4.3. DAC-ELISA

To confirm the absence of CMV in the symptomatic samples, direct antigen-coated ELISA (DAC-ELISA) was carried out following the protocol by Hobbs et al. (1987) [26]. A total of 500 mg of healthy and of infected leaf samples were ground in coating buffer containing 2% polyvinylpyrrolidone (PVP), and centrifuged at 10,000 rpm for 10 min. A total of 100 μL of extract from the samples was dispensed into wells of the polystyrene ELISA plate (Greiner Bio-One India Pvt. Ltd., Sector 62, Noida, India). The plate was incubated for 1 h at 37 °C and washed three times by flooding the wells with PBS-T for about 3 min. Then, 100 μL of blocking solution (Bovine Serum Albumin, 1%, *w*/*v*) was added to each well and incubated for 1 h at 37 °C to block polystyrene well-reactive surfaces. A total of 100 μL of CMV-specific polyclonal antiserum developed earlier in our laboratory [27], at 1:4000 dilution, was added to each well, and incubated for 1 h at 37 °C. After washing, 100 µL anti-rabbit immunoglobulin alkaline phosphatase (1:30,000, universal conjugate, Sigma, Burbank, CA, USA) was added and incubated for 1 h. Then, 100 µL freshly prepared PNPP substrate (p-nitrophenyl phosphate-PNPP, Sigma, USA), diluted with PNPP substrate buffer (1 mg PNPP tablet/mL substrate buffer), was dispensed to each well (5 mg PNPP) and incubated for 20–30 min. The intensity of colour in each well was measured at 405 nm using an ELISA reader (BIO-TEK instruments, Winooski, VT, USA).

### 4.4. Isolation of RNA and Reverse Transcriptase PCR (RT-PCR)

The total RNA was extracted from both healthy and symptomatic tomato leaves using the TRIzol reagent (Thermo Fisher Scientific, Waltham, MA, USA) following the manufacturer’s protocol. Subsequently, cDNA was synthesised from the isolated RNA using the RevertAid™ First Strand cDNA Synthesis Kit (Thermo Fisher Scientific, Waltham, MA, USA). After adding 1 µg of template RNA, the virus-specific reverse primer (10 picomole) was added, followed by heating at 65 °C for 5 min and then quickly chilling on ice. After a quick spin, 2 µL of 5× reaction buffer, 1 µL of 10 mM dNTP mix, 0.5 µL of Ribolock RNase Inhibitor (20 U/µL), and 0.5 µL of M-MuLV reverse transcriptase (20 U/µL) were added to the mixture. The mixture was incubated at 42 °C for 60 min followed by 70 °C for 5 min. The cDNAs synthesised were used as templates for PCR amplification using a universal tobamovirus primer, Tob Uni1 and Tob Uni2 [28], and primers specific to CMV, including the shoestring strain of CMV, TMV, ToMV, and ToBRFV (Table S2). The PCR-amplified product was then analysed by 1% agarose gel electrophoresis followed by gel documentation.

### 4.5. Cloning of PCR Amplicon

The RT-PCR-amplified product using universal tobamovirus genus-specific primer was purified from the agarose gel using the QIAquick^®^ Gel Extraction Kit and QIAquick^®^ PCR& Gel Clean-up Kit (QIAGEN) following the manufacturer’s protocol. The eluted product was ligated into TA cloning vector pCR™2.1 (Invitrogen by Thermo Fisher Scientific, USA), and the ligated product was used to transform DH5α cells of *E. coli*. The recombinant colonies were identified by colony PCR and restriction digestion. The confirmed clone, Tob-TA-1, was sequenced following the Sanger sequencing method through out-sourcing. 

### 4.6. Sequence Analysis

The sequence was assembled using the BioEdit Sequence Alignment Editor Version 7.2 [29]. After obtaining the sequence, two sets of primers were designed to specifically amplify the coat protein (CP) and movement protein (MP) genes of ToMMV by aligning multiple ToMMV sequences obtained from GenBank. These genes were further cloned and sequenced. CP and MP sequences of other closely related sequences were retrieved from GenBank, and multiple sequence alignment analyses were carried out using the clustalW algorithm of MEGA-X version 10.2.6 [30]. From the nucleotide sequences of the CP and MP genes, the amino acid sequences of the corresponding proteins were deduced using the Expasy server. A sequence identity matrix was generated for both CP and MP genes at the nucleotide and amino acid level using the BioEdit Sequence Alignment Editor Version 7.2 [28]. The phylogenetic tree was constructed using the neighbour-joining method with 1000 bootstrap replicates. 

### 4.7. Partial Purification of Virus 

The virus from the infected field samples was purified from at least 5 g of systemically infected tomato leaves. For this purpose, fresh plant leaf tissues showing symptoms were harvested after removing major ribs and stems and ground with liquid nitrogen in a mortar and pestle to a fine powder. The powdered tissue was homogenised with 0.1 M phosphate buffer pH 7.0 (7.5 mL for 2.5 g leaf tissue) containing 1% beta-mercaptoethanol. The homogenate was passed through two layers of muslin cloth. Then, the extract was clarified by centrifugation at 10,000 rpm for 20 min at 4 °C and the supernatant was collected. The supernatant was mixed with a 0.7 volume of chloroform and n-butanol (1:1) with continuous stirring for 15 min, and the mixture was then centrifuged at 10,000 rpm for 5 min at 4 °C. After taking out the clear aqueous layer, polyethylene glycol (PEG) 6000 (4% *w*/*v*) and NaCl (0.2 M) was added to it. The mixture was then stirred at 4 °C to dissolve the PEG and NaCl, and was kept for incubation overnight. The precipitate was collected by centrifugation at 10,000 rpm for 15 min at 4 °C and the pellet was re-suspended in 0.01 M phosphate buffer, pH 7.0 (0.5ml/g leaf tissue). The suspension was clarified by centrifugation at 10,000× *g* for 15 min at 4 °C. Then, the milky white supernatant containing virus particles was collected. Purified virion preparations, directly or after 10-fold dilution, were placed on coated carbon grids stained with 2% uranyl acetate and visualised through transmission electron microscopy (TEM).

### 4.8. Mechanical Transmission of Partially Purified Virus

Purified virion preparation after 20-fold dilution was mechanically inoculated to five plants of tomato cv. Pusa Ruby using 0.1 M phosphate buffer (pH-7.0) containing 0.2% β-mercaptoethanol. The diluted purified virion preparation was gently rubbed using cotton earbuds on the leaves of test plants pre-dusted with celite (diatomaceous earths). After 5 min, the leaves were washed with distilled water. Symptoms were observed regularly. The presence of the virus in the inoculated plants was tested by reverse transcriptase PCR using primers designed from the coat protein gene (AR47F, AR48R) (Table S2).

### 4.9. Evaluation of Response of the Virus on Different Tomato Cultivars

Purified virion preparation after 20-fold dilution was mechanically inoculated to different tomato cultivars available on the market, such as Cherry tomato, Narendra 2, Indus 32, Haemshona, Pusa Rohini, S-21, Sampurna, S-22, Sona, and Utkrisht, following the same protocol mentioned earlier in Section 4.8. For each of the test cultivars, five plants were inoculated. Then, the symptoms were observed and recorded at 7, 14, and 28 days post-inoculation. The presence of the virus was tested by reverse transcriptase PCR using primers designed from the coat protein gene (AR47F, AR48R) (Table S2).

### 4.10. Host Range Study 

A host range study was conducted to know the infectivity of the causal virus to other host plants. Plant species under the family *Solanaceae* include chilli cv. Pusa Sadabahar (*Capsicum annuum*), brinjal cv. Pusa Hara Baingan-1 (*Solanum melongena*), and tobacco (*Nicotiana tabacum* cv. Chama, *Nicotiana glutinosa*, *Nicotiana rustica* cv. Dharla, and *Nicotiana benthamiana*); plant species under the family *Cucurbitaceae* include cucumber cv. Swarna Sheetal (*Cucumis sativus*), sponge gourd cv. Dhanshree (*Luffa cylindrica*), summer squash cv. Early Round Desi (*Cucurbita pepo*), muskmelon cv. Bond (*Cucumis melo*), pumpkin cv. Desi Round (*Cucurbita maxima*), watermelon cv. Sugarbaby (*Citrullus lanatu*), and bottle gourd cv. Pusa Naveen (*Lagenaria siceraria*). Five plants each for these test species were raised in an insect-proof growth chamber. Then, the seedlings were mechanically inoculated with purified virion preparations at 20-fold dilution, as described earlier in Section 4.8. The presence of the virus was tested by reverse transcriptase PCR using primers designed from the coat protein gene (AR47F, AR48R) (Table S2).

### 4.11. Seed Transmission

To determine the seed transmissibility of the virus, seeds from infected tomato cv. Pusa Ruby plants were collected and sown in disposable pots. Fifty germinated seedlings were maintained and regularly checked for the emergence of symptoms under controlled conditions in the growth chamber. The presence of the virus was tested by reverse transcriptase PCR using primers designed from the coat protein gene (AR47F, AR48R) (Table S2).

## 5. Conclusions

In conclusion, this study represents the first footprint of ToMMV in India causing shoestring symptoms in tomato plants under natural conditions. The virus is highly contagious and can be readily transmitted through mechanical means and seeds with a low transmission rate. Its potential to inflict substantial damage in leading commercial cultivars of tomato and chilli poses a risk to the cultivation of these vegetables in India. Additional surveys are required to establish the frequency and spread of ToMMV-Tss in the country’s tomato-growing belts. Investigating the genetic mechanisms of disease development and plant defence against ToMMV-Tss in the future will be necessary to develop resistance in the tomato crop.

## Figures and Tables

**Table 1:** Percent sequence identity of the coat protein and movement protein sequences of ToMMV-Tss with other isolates of ToMMV obtained from GenBank and used in phylogenetic analysis.

Sl. No.	Accession No. and Virus Isolates	Host	Location	Percent Sequence Identity			
				CP		MP	
				Nt	Aa	Nt	Aa
1	MH381817.1 ToMMV isolate HN	*Solanum melongena*	China	99.5	99.3	99.0	99.2
2	MG171192.1 ToMMV isolate Hainan	*Solanum lycopersicum*	China	99.5	99.3	99.0	99.2
3	KX898033.1 ToMMV isolate SC13-05	*Solanum lycopersicum*	USA	99.5	99.3	99.1	99.2
4	OK334224.1 ToMMV isolate ToMMV 83	*Solanum lycopersicum*	Viet Nam	99.5	99.3	99.3	99.6
5	ON987481.1 ToMMV isolate NPPO-NL 41979438	*Solanum lycopersicum*	France	99.3	99.3	99.3	99.6
6	MW373515.1 ToMMV isolate Shandong	*Solanum lycopersicum*	China	99.3	98.7	99.3	99.6
7	MZ713256.1 ToMMV isolate NVWA36783860	*Solanum lycopersicum*	China	99.3	99.3	99.3	99.6
8	KF477193.1 ToMMV isolate MX5	*Solanum lycopersicum*	Mexico	99.3	99.3	99.1	99.2
9	MH128145.1 ToMMV isolate CpB1	*Solanum lycopersicum*	S. America	99.1	99.3	98.8	99.2
10	KR824951.1 ToMMV isolate TiLhaLJ	*Solanum lycopersicum*	China	99.1	99.3	99.3	99.6
11	KP202857.1 ToMMV isolate 10-100	*Solanum lycopersicum*	USA	99.1	99.3	99.0	99.2
12	MN853592.1 ToMMV isolate LN	*Solanum lycopersicum*	China	99.1	99.3	98.6	98.5
13	KR824950.1 ToMMV isolate YYMLJ	*Solanum lycopersicum*	China	98.9	99.3	99.3	99.6
14	ON146334.1 ToMMV MZWD	*Pisum sativum*	China	98.9	99.3	99.2	99.2
15	OK180812.1 ToMMV isolate GD-2020	*Capsicum annuum*	China	98.9	99.3	98.6	98.8
16	KY912162.1 ToMV isolate SL-1	*Solanum lycopersicum*	Slovakia	85.6	90.5	76.9	76.4
17	MW848529.1 ToMV isolate NGSTPS18/DSMZ PV-0135	*Solanum lycopersicum*	Germany	85.6	90.5	76.9	76.4
18	MH393623.1 ToMV isolate ToMV DonDoung	*Capsicum annuum*	Republic of Korea	85.4	90.5	77.3	76.4
19	LC650928.1 ToMV ToMV-KMT RNA	*Solanum lycopersicum*	Japan	85.4	91.1	77.3	76.4
20	OL652661.1 ToMV isolate NVWA5785660	*Solanum lycopersicum*	Netherlands	85.4	91.1	77.1	76.4
21	MW012409.1 ToMV isolate DTT	*Solanum lycopersicum*	Republic of Korea	85.4	90.5	77.3	76.4
22	MF002483.1 ToMV strain SX1	*Solanum lycopersicum*	China	85.2	90.5	77.1	76.1
23	KY967227.1 ToMV isolate YT	*Solanum lycopersicum*	China	85.2	91.1	77.1	76.4
24	PP915779.1 ToMV isolate Pi20	*Eriobotrya japonica*	China	85.2	90.5	77.4	76.8
25	MZ388458.1 ToMV isolate ToMV-WF	*Solanum lycopersicum*	China	85.2	91.1	76.9	76.1
26	EF392659.1 TMV strain pet-TW	*Petunia hybrida*	Taiwan	76.8	82.3	59.1	65.6
27	MW051785.1 TMV isolate DSMZ PV-0171		Germany	76.4	81.1	59.4	64.9
28	OQ633220.1 ToBRFV isolate 402275 1	*Solanum lycopersicum*	Belgium	75.6	79.8	72.6	72.3
29	OR792460.1 ToBRFV isolate ToBRFV-SD	*Solanum lycopersicum*	China	75.6	80.5	72.8	72.3
30	MN882050.1 ToBRFV isolate 39941596 B-2	*Solanum lycopersicum*	Netherlands	79.7	84.9	72.7	72.3
31	OM892685.1 ToBRFV isolate S27	*Solanum lycopersicum*	USA	79.7	84.9	72.7	72
32	D12505.1 CGMMV	*Cucumis sativus*	Japan	44.9	32.9	42.2	28.3

**Table 2:** Responses of different solanaceous hosts upon mechanical inoculation of partially purified ToMMV-Tss.

Name of Test Hosts	Symptoms Observed	Frequency of Symptoms	Days Taken for Symptom Expression	RT-PCR Frequency
Tomato (*Solanum lycopersicum*)				
cv. S-21	Systemic mosaic, shoestring	5/5	4–5	5/5
cv. Indus 32	Systemic mosaic, shoestring	5/5	7–10	5/5
cv. Narendra 2	Systemic mosaic, shoestring	5/5	7–8	5/5
cv. Haemshona	No symptoms	0/5	-	0/5
cv. Pusa Rohini	Systemic mosaic, shoestring	5/5	7–10	5/5
cv. S-22	Systemic mosaic, shoestring	5/5	4–5	5/5
cv. Cherry tomato	Systemic mosaic, shoestring	5/5	7–10	5/5
cv. Sampurna	No symptoms	0/5	-	0/5
cv. Utkrisht	Systemic mosaic, shoestring	5/5	4–5	5/5
cv. Sona	Systemic mosaic, shoestring	5/5	4–5	5/5
Brinjal (*Solanum melongena*) cv. Pusa Hara Baingan	No symptoms	0/5	-	5/5
Chilli (*Capsicum annuum*) cv. Pusa Sadabahar	Systemic green mosaic	5/5	90–95	5/5
*Nicotiana tabacum* cv. Chama	No symptoms	0/5	-	0/5
*Nicotiana glutinosa*	Necrotic local lesion, marginal upward rolling of systemic leaves	5/5	6–7	5/5
*Nicotiana rustica* cv. Dharla	Necrotic local lesion, puckering of systemic leaves	5/5	6–7	5/5
*Nicotiana benthamiana*	Downward curling of leaves, puckering and blistering of systemic leaves, severely stunted appearance	5/5	3–4	5/5

## Data Availability

All data generated or analysed during this study are included in this published article and its Supplementary Materials.

## Supplementary Materials

The following supporting information can be downloaded at https://www.mdpi.com/article/10.3390/plants13192811/s1: Figure S1. Confirmation of absence of CMV and other tobamovirus species in shoe-string symptomatic tomato plants in India through direct antibody coated ELISA (DAC-ELISA) and reverse-transcriptase PCR (RT-PCR). (a) DAC-ELISA for detection of CMV in symptomatic tomato cv. Pusa Ruby plants. Yellow color: Positive; No color: Negative; S1 to S5: Shoe-string disease affected samples; Positive control: CMV (partially purified). (b) Agarose gel (1%) electrophoresis of RT-PCR amplified products obtained using CMV specific primer, CMV shoe-string strain (Lucknow region) specific primer (c), CMV shoe-string strain (New Delhi region) specific primer (d), ToMV specific primer (e), TMV specific primer (f), and ToBRFV specific primer (g); Figure S2. RT-PCR amplification of complete coat protein (CP) and movement protein (MP) genes of shoe-string isolate of ToMMV. (a) Agarose gel (1%) electrophoresis of RT-PCR amplified products obtained using CP specific primers and MP specific primers (b). M: molecular weight marker; 1: negative control; 2 to 5: test samples; Figure S3. Representative transmission electron micrograph of partially purified ToMMV using 2% uranyl acetate (negative stain) under 80000X magnification showing presence of only rigid rod shaped particle (~300 x 18 nm); Figure S4. Response of different hosts under family Cucurbitaceae upon mechanical inoculation of partially purified shoe-string isolate of ToMMV. (a) Absence of any symptom in Cucumber cv. Swarna Sheetal, Sponge gourd cv. Dhanshree, Summer squash cv. Early Round Desi, Muskmelon cv. Bond, Pumpkin cv. Desi Round, Watermelon cv. Sugarbaby and Bottle Gourd cv. Pusa Naveen. (b) Agarose gel (1%) electrophoresis of RT-PCR amplified coat protein gene of the shoe-string isolate of ToMMV using specific primers. Absence of amplified product in all the tested cucurbitaceous hosts. Lane 1: healthy control, Lane 2: Positive control, Lane 3-9: Cucumber, Sponge gourd, Summer squash, Muskmelon, Pumpkin, Watermelon and Bottle Gourd, respectively; Table S1. Data representing DAC-ELISA results of symptomatic tomato plants cv. Pusa Ruby using polyclonal antisera against CMV; Table S2. List of Primers used in the study.
