# Supplementary material for: Biological and Molecular Characterization of a New Isolate of Tomato Mottle Mosaic Virus Causing Severe Shoestring and Fruit Deformities in Tomato Plants in India
Source: Plants (Basel). 2024 Oct 8;13(19):2811. doi: 10.3390/plants13192811 (PMC11478595; doi:10.3390/plants13192811)
Supplement: Supplementary file 1 [file plants-13-02811-s001.zip › Original gel images.pptx]

## Slide 1
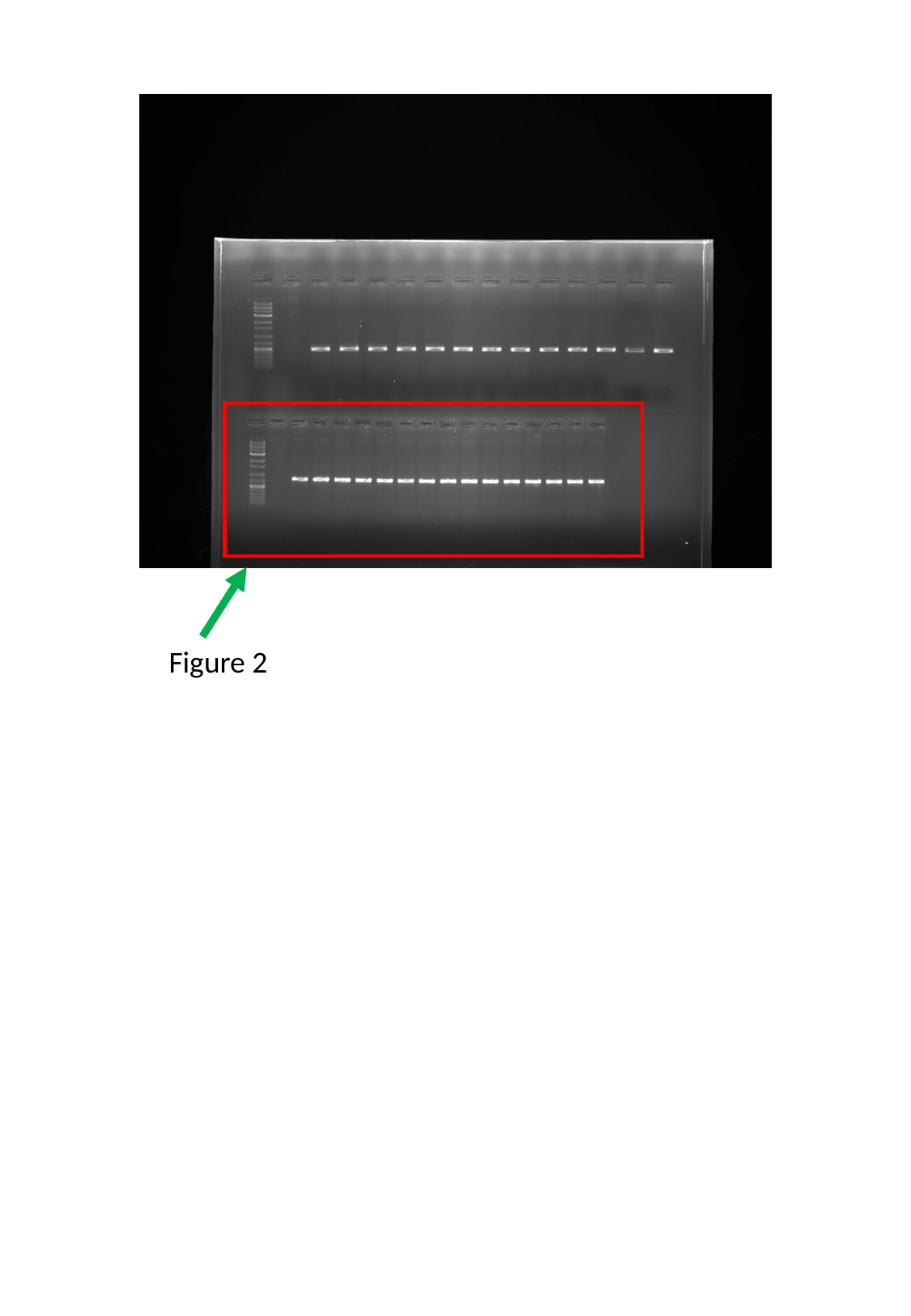

Figure 2

## Slide 2
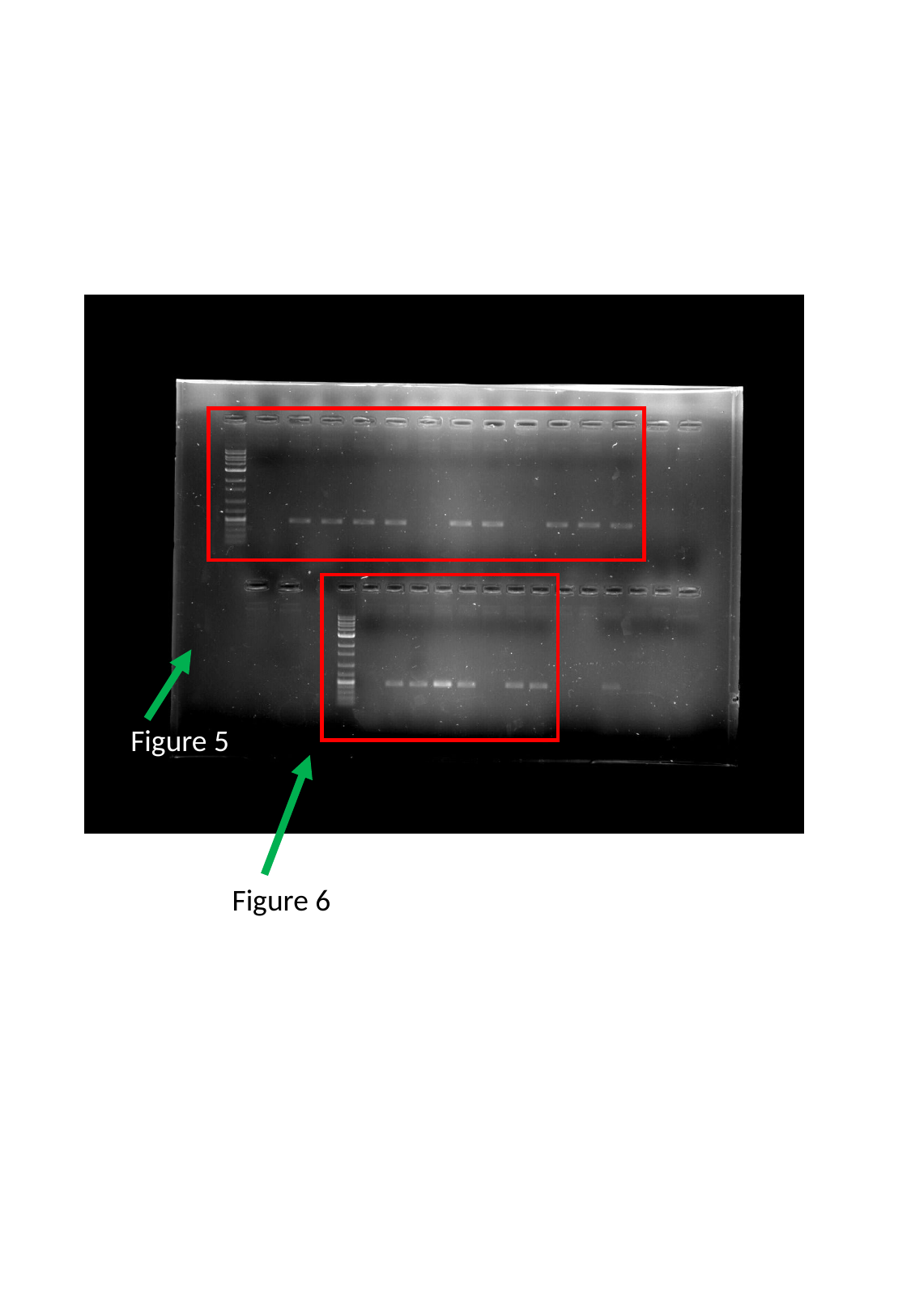

Figure 5
Figure 6

## Slide 3
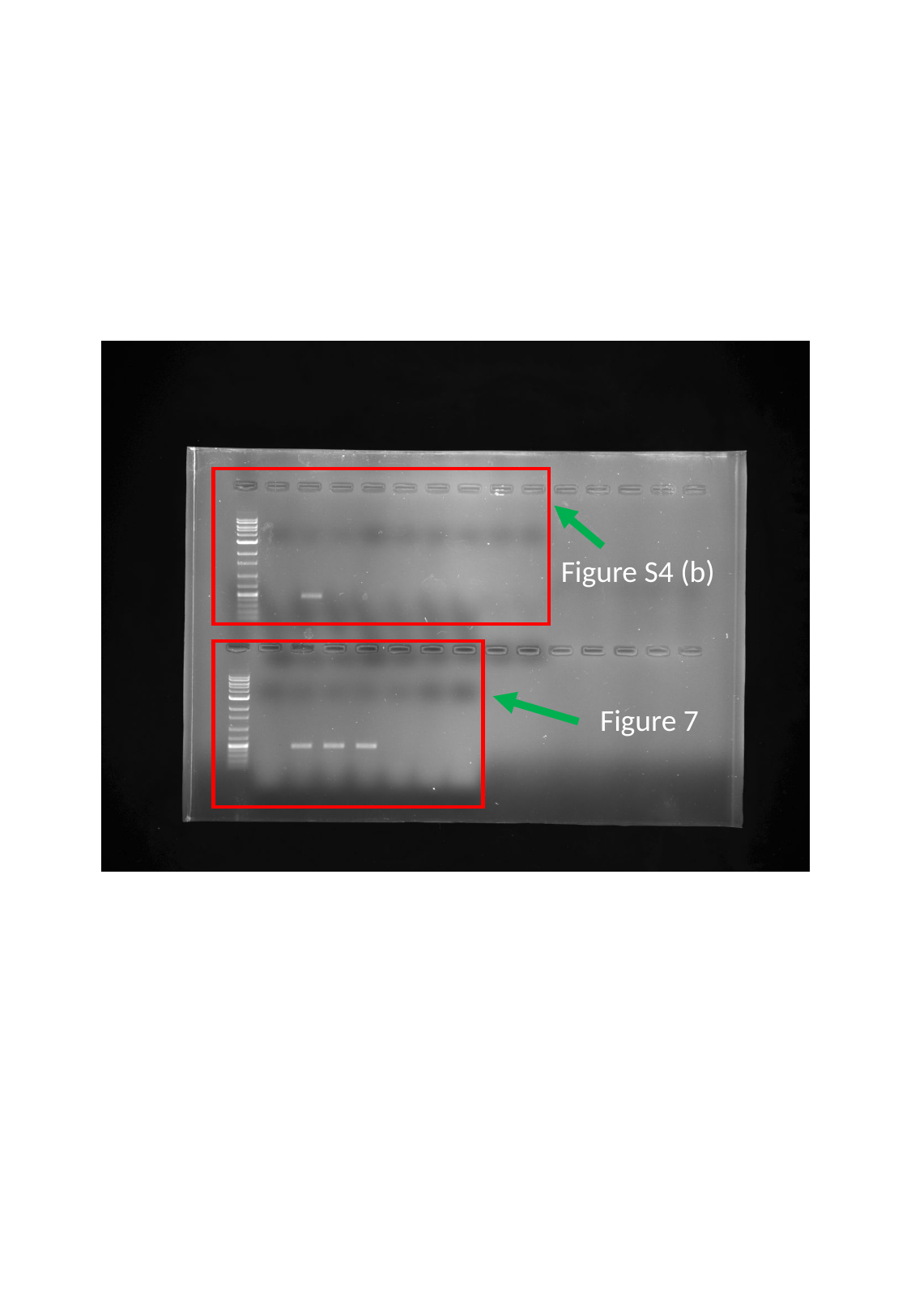

Figure S4 (b)
Figure 7

## Slide 4
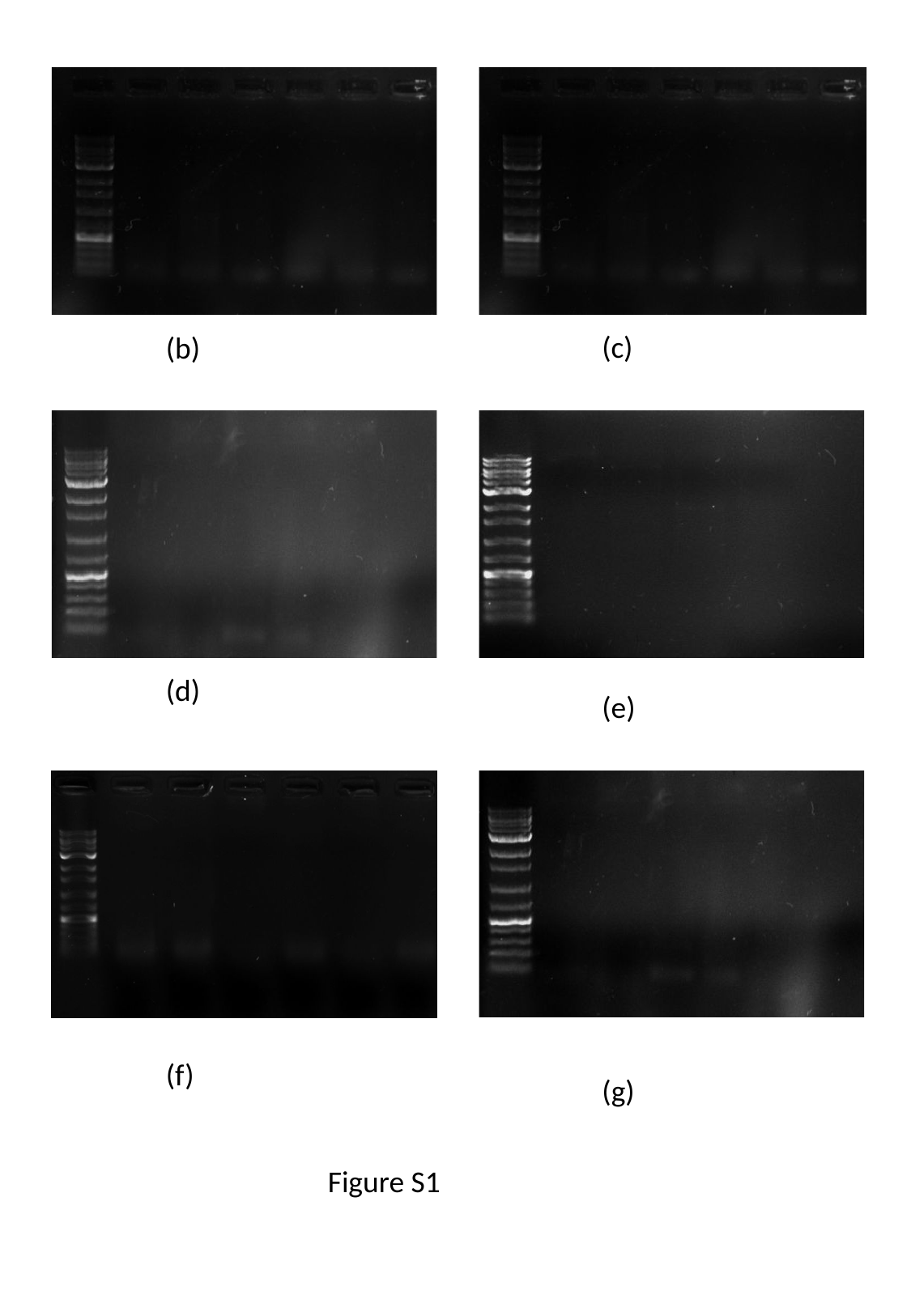

(c)
(b)
(d)
(e)
(f)
(g)
Figure S1

## Slide 5
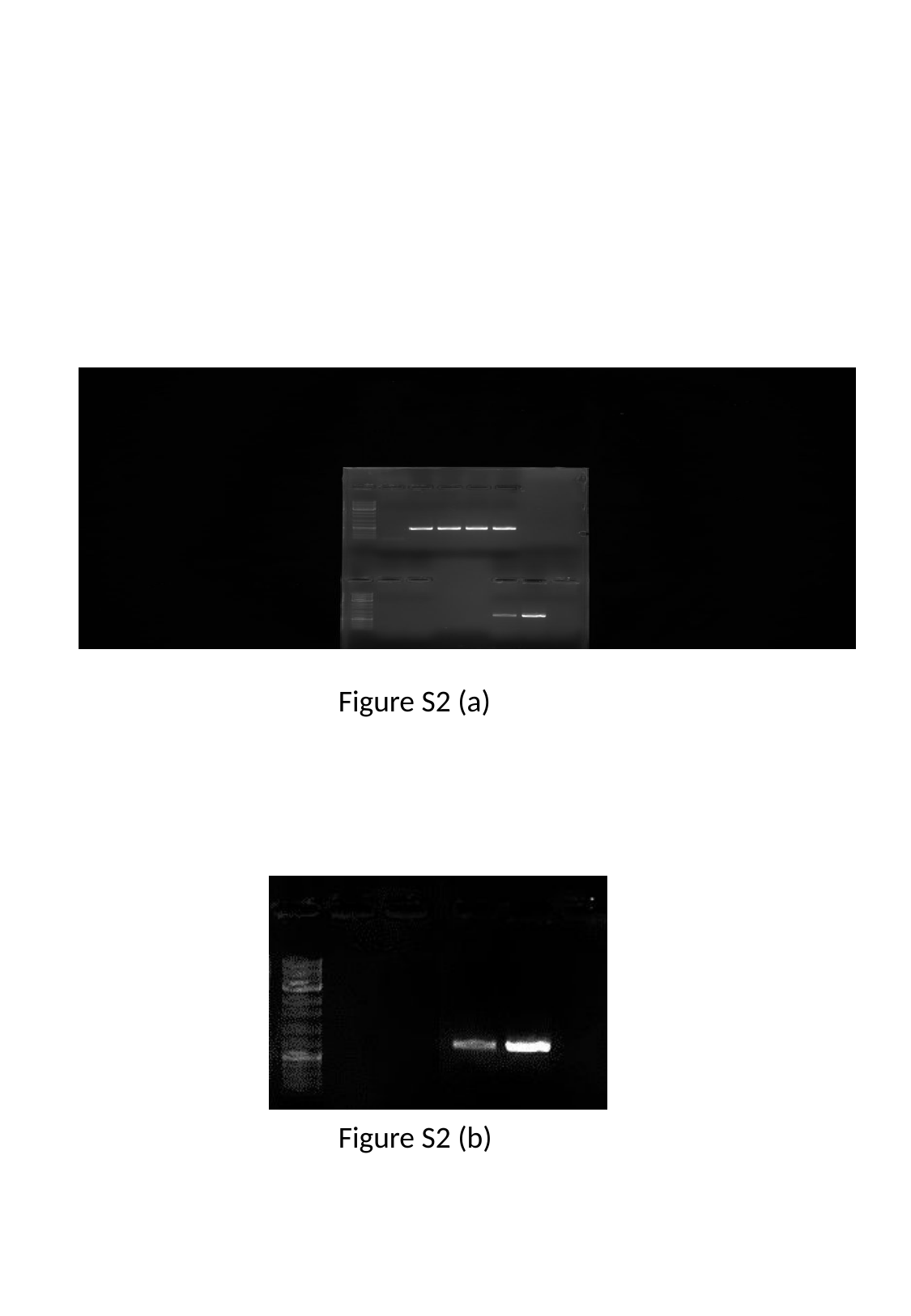

Figure S2 (a)
Figure S2 (b)
Figure S2 (a)
