# Supplementary material for: Biological and Molecular Characterization of a New Isolate of Tomato Mottle Mosaic Virus Causing Severe Shoestring and Fruit Deformities in Tomato Plants in India
Source: Plants (Basel). 2024 Oct 8;13(19):2811. doi: 10.3390/plants13192811 (PMC11478595; doi:10.3390/plants13192811)
Supplement: Supplementary file 1 [file plants-13-02811-s001.zip › Supplementary Figures/Legends Supplimentary Figure.pdf]

Figure S1. Confirmation of absence of CMV and other tobamovirus species in shoe-string symptomatic tomato plants in India through direct antibody coated ELISA (DAC-ELISA) and reverse-transcriptase PCR (RT-PCR). (a) DAC-ELISA for detection of CMV in symptomatic tomato cv. Pusa Ruby plants. Yellow color: Positive; No color: Negative; S1 to S5: Shoe-string disease affected samples; Positive control: CMV (partially purified). (b) Agarose gel (1%) electrophoresis of RT-PCR amplified products obtained using CMV specific primer, CMV shoe-string strain (Lucknow region) specific primer (c), CMV shoe-string strain (New Delhi region) specific primer (d), ToMV specific primer (e), TMV specific primer (f), and ToBRFV specific primer (g).

Figure S2. RT-PCR amplification of complete coat protein (CP) and movement protein (MP) genes of shoe-string isolate of ToMMV. (a) Agarose gel (1%) electrophoresis of RT-PCR amplified products obtained using CP specific primers and MP specific primers (b). M: molecular weight marker; 1: negative control; 2 to 5: test samples

Figure S3. Representative transmission electron micrograph of partially purified ToMMV using 2% uranyl acetate (negative stain) under 80000X magnification showing presence of only rigid rod shaped particle (~300 x 18 nm).

Figure S4. Response of different hosts under family Cucurbitaceae upon mechanical inoculation of partially purified shoe-string isolate of ToMMV. (a) Absence of any symptom in Cucumber cv. Swarna Sheetal, Sponge gourd cv. Dhanshree, Summer squash cv. Early Round Desi, Muskmelon cv. Bond, Pumpkin cv. Desi Round, Watermelon cv. Sugarbaby and Bottle Gourd cv. Pusa Naveen. (b) Agarose gel (1%) electrophoresis of RT-PCR amplified coat protein gene of the shoe-string isolate of ToMMV using specific primers. Absence of amplified product in all the tested cucurbitaceous hosts. Lane 1: healthy control, Lane 2: Positive control, Lane 3-9: Cucumber, Sponge gourd, Summer squash, Muskmelon, Pumpkin, Watermelon and Bottle Gourd , respectively
