# Supplementary material for: Biological and Molecular Characterization of a New Isolate of Tomato Mottle Mosaic Virus Causing Severe Shoestring and Fruit Deformities in Tomato Plants in India
Source: Plants (Basel). 2024 Oct 8;13(19):2811. doi: 10.3390/plants13192811 (PMC11478595; doi:10.3390/plants13192811)
Supplement: Supplementary file 1 [file plants-13-02811-s001.zip › Supplementary Tables/Table S1.pdf]

Table S1. Data representing DAC-ELISA results of symptomatic tomato plants cv. Pusa Ruby using polyclonal antisera against CMV

Plant Sample	Average OD* value				Inference
	Test Sample	Healthy control	Buffer control	Positive Control	
Plant Sample 1 (S1)	0.0358	0.0223	0.0083	2.3862	**Negative**
Plant Sample 2 (S2)	0.0476	0.0223	0.0083	2.3862	**Negative**
Plant Sample 3 (S3)	0.0394	0.0223	0.0083	2.3862	**Negative**
Plant Sample 4 (S4)	0.0455	0.0223	0.0083	2.3862	**Negative**
Plant Sample 5 (S5)	0.0349	0.0223	0.0083	2.3862	**Negative**
