# Supplementary material for: Biological and Molecular Characterization of a New Isolate of Tomato Mottle Mosaic Virus Causing Severe Shoestring and Fruit Deformities in Tomato Plants in India
Source: Plants (Basel). 2024 Oct 8;13(19):2811. doi: 10.3390/plants13192811 (PMC11478595; doi:10.3390/plants13192811)
Supplement: Supplementary file 1 [file plants-13-02811-s001.zip › Supplementary Tables/Table S2.pdf]

Table S2. List of Primers used in the study

Sl. No	Primer Name	Primer Sequences *(5'-3')	Amplicon Size (bp)	Primers specific for	Targeting ORF	References
1	Tob-Uni1<br>Tob-Uni2	GTYGTTGATGAGTTCGTGGA<br>ATTTAAGTGGAGGGAAAACCACT	801	Tobamovirus genus specific	CP+ partial MP	Pappu and Druffel, 2007
2	AR09F<br>AR10R	ATGGACAAATCTGAATCAACCRGTG<br>GACTGGGAGCACYCCAGAYG	657	Cucumber mosaic virus (CMV)	CP	Designed in this study
3	CMV-Ts-F<br>CMV-Ts-R	ATGGACAAATCTGAATCAACCAGTGC<br>AACTGGGAGCACTCCAGATGTG	657	CMV shoe-string strain (Lucknow isolate)	CP	Pratap et al., 2012
4	CMV-TssIn-F<br>CMV-TssIn-R	ATGGACAAATCTGGATCTCCCAATGCTAG<br>GACTGGGAGCACCCGTGAGATAG	657	CMV shoe-string strain (New Delhi isolate)	CP	Geetanjali et al., 2011
5	ToBRFV-F<br>ToBRFV-R	CACAATCGCAACTCCATCGC<br>CAGAGGACCATTGTAAACCGG	458	Tomato brown rugose fruit virus (ToBRFV)	CP	Kavya et al., 2024
6	AR67F<br>AR68R	ATGTCTTACKCWATYACTTCTC<br>CCAASCCAGACATACTTTC	454	Tomato mosaic virus (ToMV)	CP	Designed in this study
7	AR-69F<br>AR-70R	ATGTCTTACHCAATYACWWCTCCATC<br>AGATGCAGGWSCAGARGWCCA	480	Tobacco mosaic virus (TMV)	CP	Designed in this study
8	AR47F<br>AR48R	ATGTCTTACGCTATTACTTCTCCGTC<br>GGACGCTGGCGCAGAAG	477	Tomato mottle mosaic virus (ToMMV)	CP	Designed in this study
9	AR51F<br>AR52R	ATGGCTCTAACTGTTAGTGGTAAAG<br>ATACGAATCAGATCCCGCGAC	804	Tomato mottle mosaic virus (ToMMV)	MP	Designed in this study
