# Supplementary figures and images for: Biological and Molecular Characterization of a New Isolate of Tomato Mottle Mosaic Virus Causing Severe Shoestring and Fruit Deformities in Tomato Plants in India
Source: Plants (Basel). 2024 Oct 8;13(19):2811. doi: 10.3390/plants13192811 (PMC11478595; doi:10.3390/plants13192811)

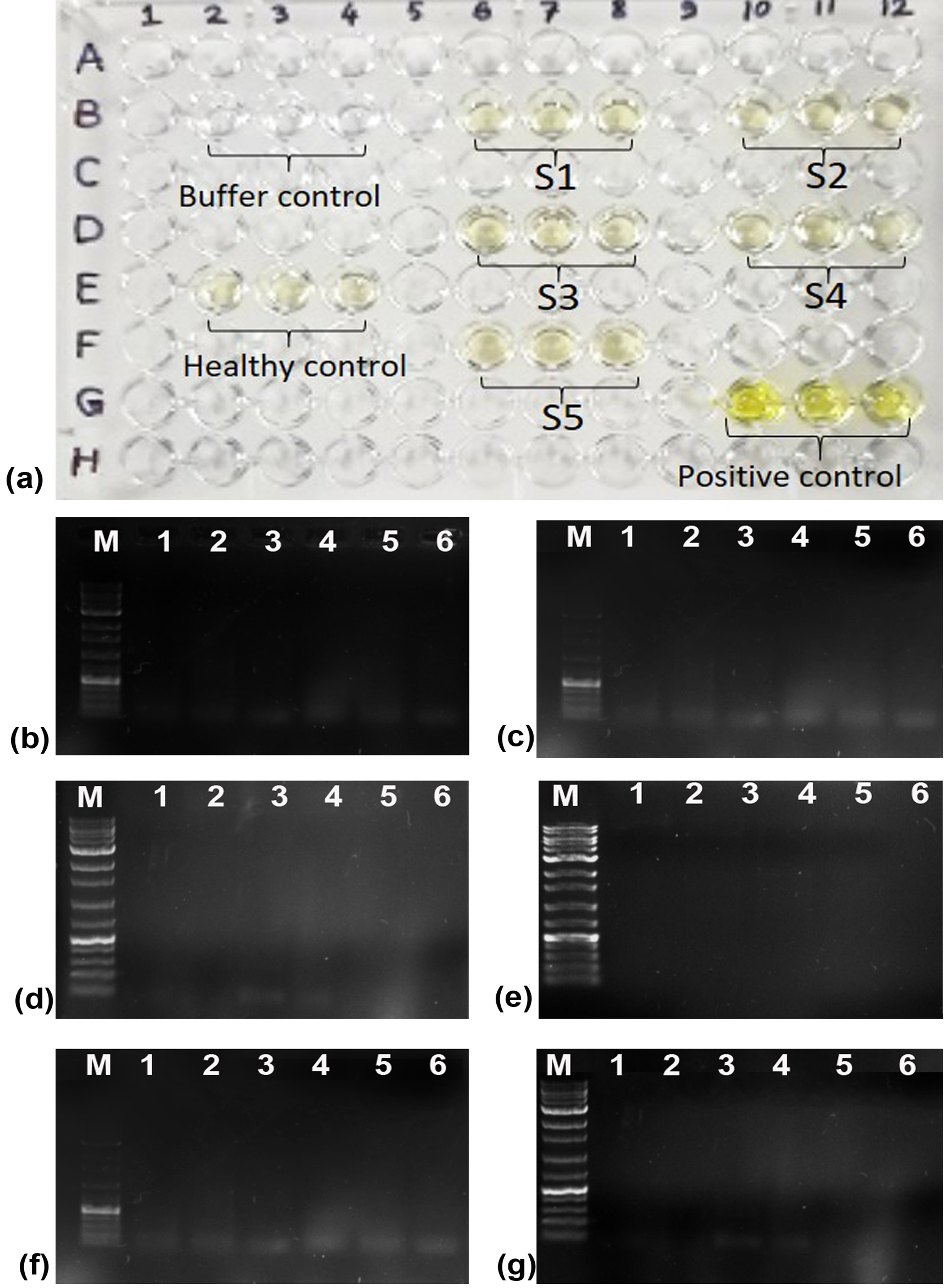

Supplement: Supplementary file 1 [file plants-13-02811-s001.zip › Supplementary Figures/Figure S1.jpg]

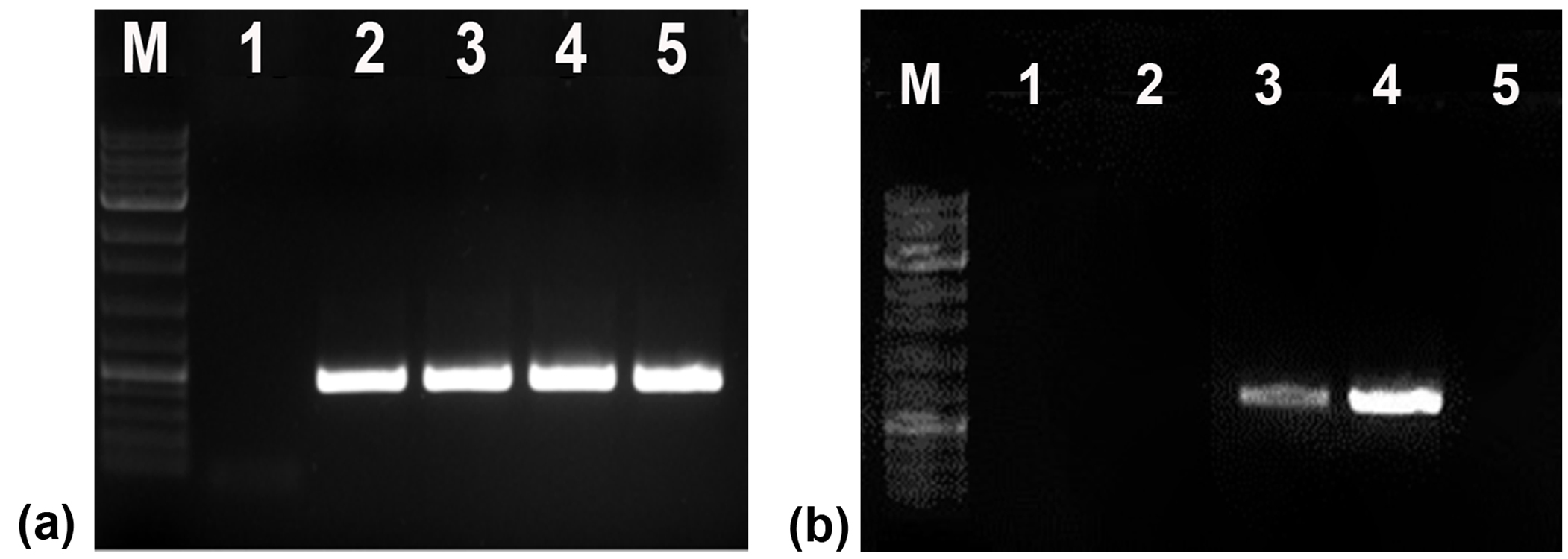

Supplement: Supplementary file 1 [file plants-13-02811-s001.zip › Supplementary Figures/Figure S2.jpg]

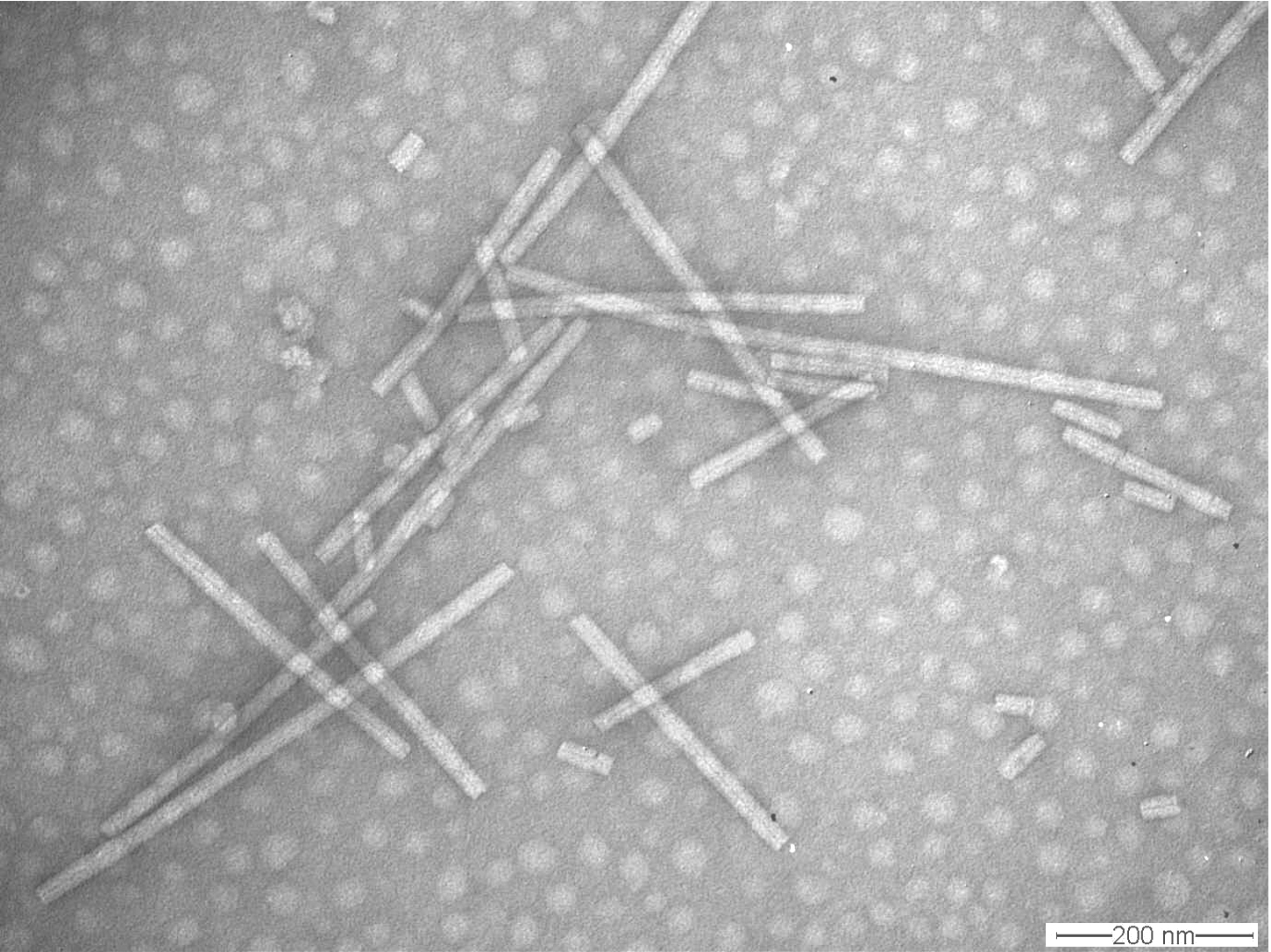

Supplement: Supplementary file 1 [file plants-13-02811-s001.zip › Supplementary Figures/Figure S3.jpg]

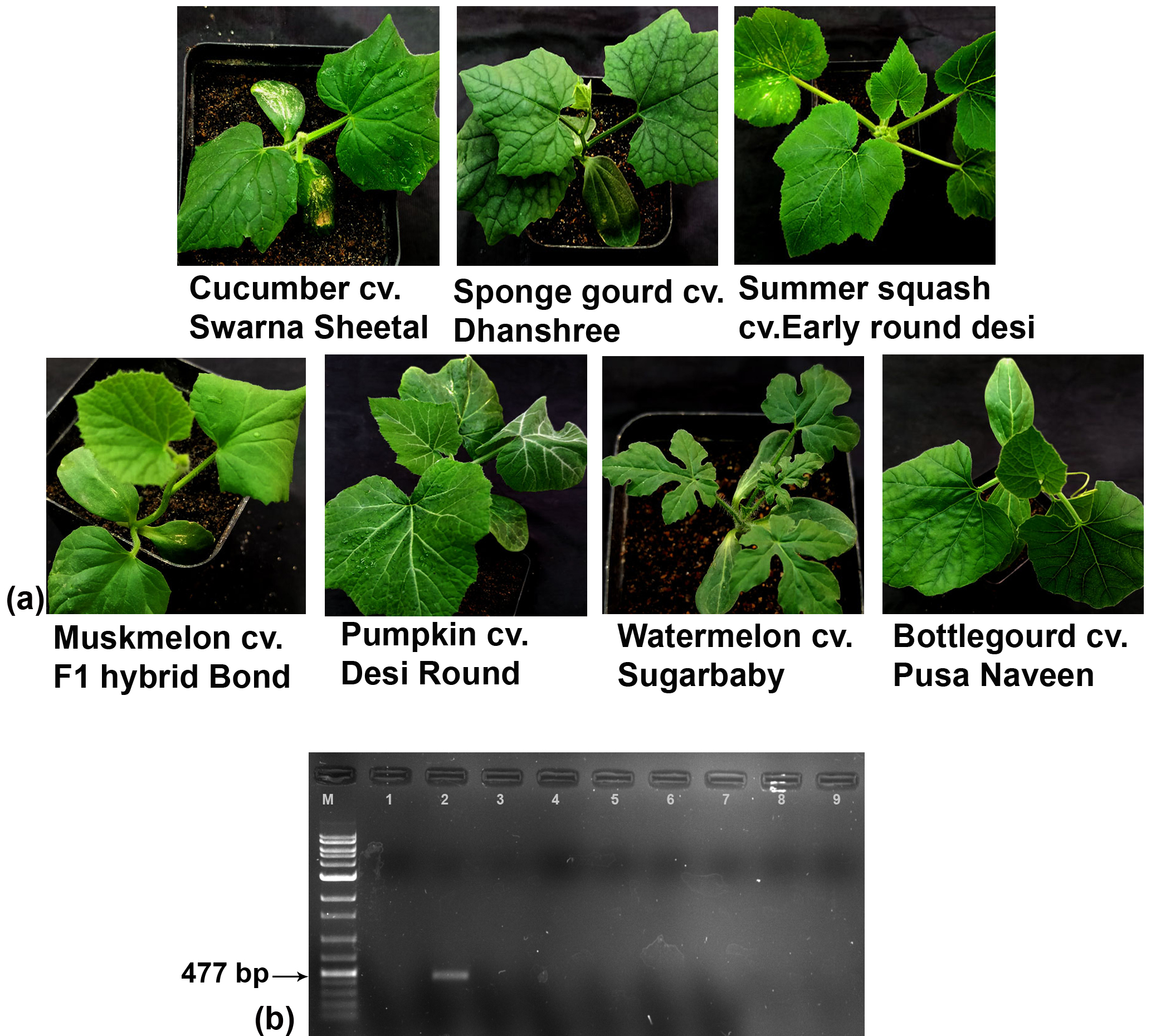

Supplement: Supplementary file 1 [file plants-13-02811-s001.zip › Supplementary Figures/Figure S4.jpg]
